# Inceptor facilitates acrosomal vesicle formation in spermatids and is required for male fertility

**DOI:** 10.3389/fcell.2023.1240039

**Published:** 2023-08-24

**Authors:** Sara Bilekova, Balma Garcia-Colomer, Alberto Cebrian-Serrano, Silvia Schirge, Karsten Krey, Michael Sterr, Thomas Kurth, Stefanie M. Hauck, Heiko Lickert

**Affiliations:** ^1^ Helmholtz Center Munich, German Research Center for Environmental Health GmbH, Institute of Diabetes and Regeneration Research, Neuherberg, Germany; ^2^ German Center for Diabetes Research (DZD), Neuherberg, Germany; ^3^ School of Medicine, Technical University of Munich, Munich, Germany; ^4^ Helmholtz Center Munich, Institute for Diabetes and Obesity, Neuherberg, Germany; ^5^ Institute of Virology, Technical University of Munich, Munich, Germany; ^6^ Center for Molecular and Cellular Bioengineering (CMCB), Technology Platform, Core Facility Electron Microscopy and Histology, Dresden University of Technology, Dresden, Germany; ^7^ Metabolomics and Proteomics Core, Helmholtz Center Munich, German Research Center for Environmental Health GmbH, Munich, Germany

**Keywords:** acrosome, differentiation, male fertility, spermatogenesis, vesicle, localization

## Abstract

Spermatogenesis is a crucial biological process that enables the production of functional sperm, allowing for successful reproduction. Proper germ cell differentiation and maturation require tight regulation of hormonal signals, cellular signaling pathways, and cell biological processes. The acrosome is a lysosome-related organelle at the anterior of the sperm head that contains enzymes and receptors essential for egg-sperm recognition and fusion. Even though several factors crucial for acrosome biogenesis have been discovered, the precise molecular mechanism of pro-acrosomal vesicle formation and fusion is not yet known. In this study, we investigated the role of the insulin inhibitory receptor (inceptor) in acrosome formation. Inceptor is a single-pass transmembrane protein with similarities to mannose-6-phosphate receptors (M6PR). Inceptor knockout male mice are infertile due to malformations in the acrosome and defects in the nuclear shape of spermatozoa. We show that inceptor is expressed in early spermatids and mainly localizes to vesicles between the Golgi apparatus and acrosome. Here we show that inceptor is an essential factor in the intracellular transport of *trans*-Golgi network-derived vesicles which deliver acrosomal cargo in maturing spermatids. The absence of inceptor results in vesicle-fusion defects, acrosomal malformation, and male infertility. These findings support our hypothesis of inceptor as a universal lysosomal or lysosome-related organelle sorting receptor expressed in several secretory tissues.

## 1 Introduction

Murine male germ cells show a marked upregulation of over 1500 genes at the onset of meiosis and the expression of around 350 of these genes is restricted to the male germ line, many of them directly necessary for the fertility of sperm (Schultz et al., 2003). However, many testis-specific genes have been found to be dispensable, most likely due to the robustness and redundancy of the system (Miyata et al., 2016; Lu et al., 2019; Park et al., 2020). This illustrates the complexity and evolutionary necessity of the tight regulation of spermatogenesis. Even though the cell biology of spermatogenesis has been well described, the molecular mechanism and variety of involved factors remain poorly characterized.

Spermatogenesis takes place in the seminiferous tubules of the testis. The spermatogonia undergo mitosis and develop into spermatocytes. In meiotic spermatocytes of humans and mice, heavily glycosylated soluble hydrolases, zymogens, transmembrane proteins, and other acrosome-specific cargo mature through the ER-Golgi networks and are packed into proacrosomal vesicles (pro-AVs) (Anakwe, 1990; Kashiwabara et al., 1990; Escalier et al., 1991). In mice, after the completion of meiosis, spermatids develop in a 16-step process (Oakberg, 1956). In steps 1-3, pro-AVs fuse and attach to the nuclear membrane (Escalier et al., 1991; Bermudez et al., 1994). How this process is initiated and how premature fusion is inhibited, is so far unknown. Trafficking between the Golgi apparatus and the acrosome is dynamic and bidirectional, with several Golgi proteins found on acrosomes that are later retrieved (Moreno et al., 2000). In steps 3-4, F-actin forms the acroplaxome adjacent to the nuclear membrane to which keratin V is added in steps 4–5. This cytoskeletal structure forms the site of pro-AV attachment to the nuclear lamina, initiating acrosome formation (Kierszenbaum et al., 2004). Adaptor protein 1 (AP-1) has been proposed to be the clathrin-adaptor protein mediating pro-AV trafficking towards the acrosome (Kang-Decker et al., 2001). However, it is unknown if AP-1 is present at the time of fusion and how the pro-AVs merge. Clathrin and adaptins are expressed in steps 1-5, whereas syntaxins and vesicle-associated membrane proteins (VAMPs) which mediate membrane fusion, have been found to localize to the pro-AVs in steps 1-2 and later to the acrosome (Ramalho-Santos et al., 2001). The acrosomal shape has been used to describe the characteristic spermatid development stages: the Golgi phase, when the acrosomal material is located near the Golgi apparatus, the cap phase, when the acrosome grows and spreads onto the nuclear surface, the acrosomal stage, when the nucleus and acrosome elongate, and finally the maturation stage. The spermatozoon continues to mature throughout its passage through the epididymis and female reproductive tract.

The insulin inhibitory receptor (in short: inceptor, gene *Iir*, also known as Elapor1) has been previously described in the context of cancer prognosis and progression (Deng et al., 2005; Kang et al., 2015; Stinnesbeck et al., 2021; Wissmiller et al., 2023). Moreover, we have shown that inceptor desensitizes insulin and insulin-like growth factor (IGF) receptor signaling in pancreatic beta cells (Ansarullah et al., 2021). Inceptor contains an adaptor protein 2 (AP-2) sorting signal and interacts with the *µ* subunit of AP-2 (Ansarullah et al., 2021). From our most recent findings in pancreatic beta cells, we propose that inceptor is a sorting and degradation receptor found in clathrin-coated vesicles budding from the plasma membrane and *trans*-Golgi membrane that routes insulin and proinsulin to lysosomal degradation (Siehler et al., unpublished results). Additionally, inceptor has been found to regulate secretory granule formation in zymogenic cells in the stomach (Cho et al., 2022). Early spermatids also express inceptor, and interestingly, the acrosome formation process has similarities with both regulated secretion and lysosome biogenesis (Khawar et al., 2019). Therefore, we hypothesized that inceptor might play a role in acrosomal trafficking and acrosome formation.

The acrosome is a large granule at the anterior end of the sperm head essential for sperm-oocyte recognition and fusion (Khawar et al., 2019). It is a low-pH organelle that contains Golgi-derived cargo, which is either acrosome-specific or lysosome-like. Therefore, the acrosome has been proposed to be a lysosome-related organelle (Berruti et al., 2010). It contains several enzymes, zymogens, and receptors necessary for oocyte recognition. At the time of oocyte-sperm contact, the acrosome is exocytosed as the outer acrosomal membrane fuses with the plasma membrane, enabling the sperm to penetrate the *zona pellucida* around the oocyte. Then, the inner acrosomal membrane fuses with the oocyte’s plasma membrane. The acrosome reaction has been reviewed in detail (Aldana et al., 2021).

Some of the factors which play a role in insulin granule formation or secretion are also essential for acrosome formation, such as Protein interacting with C kinase 1 (PICK1), Golgi-associated PDZ- and coiled-coil motif-containing protein (GOPC), or Mysoin Va (Yao et al., 2002; Kierszenbaum et al., 2003; Varadi et al., 2005; Xiao et al., 2009; Wilhelmi et al., 2021; Andersen et al., 2022). Moreover, Golgi and lysosomal proteins are retrieved from acrosomes in pathways resembling retrieval from maturing secretory granules in neuroendocrine cells, mediated by AP-1 and STX6 (Klumperman et al., 1998). The acrosome also displays similarities with the lysosome, but it is unknown whether it can carry out lysosomal metabolic functions in sperm. Even though the acrosome contains several unique proteins, it also contains typical lysosomal proteins, such as cathepsin D and H (Moreno and Alvarado, 2006). In addition to the acrosome’s similarities to lysosomes and secretory granules, recent studies point to mitochondrial engagement in acrosome formation. Condensed mitochondria were found to act as donors to the AV, and several mitochondrial enzymes were shown to localize to the acrosome (Ren et al., 2019; 2022; Otčenášková et al., 2023). Altogether, this shows that there is a substantial gap in knowledge regarding the acrosomal origin and the molecular mechanism of its biogenesis.

Here, we demonstrate the importance of inceptor in male fertility in a whole-body inceptor knockout (KO) mouse model, showing that inceptor is essential for the formation of morphologically normal sperm. Inceptor KO spermatids are characterized by acrosomal malformation, an irregular or round nucleus, and reduced motility. We propose that inceptor is tightly involved in acrosome development in early spermatids and is directly involved in cargo transport and delivery to the forming AV.

## 2 Materials and methods

### 2.1 Mouse models

Animal experiments were carried out in compliance with the German Animal Protection Act and with the approved guidelines of the Society of Laboratory Animals (GV-SOLAS) and of the Federation of Laboratory Animal Science Associations (FELASA). Whole-body *Iir* ^*−/−*^ mice were generated from the previously described GeneTrap allele (Ansarullah et al., 2021). *Iir* ^*−/−*^: 5330417C22Rik^tm1a(EUCOMM)Hmgu^ embryonic stem cells were aggregated with CD1 morula to generate chimeric mice. For critical exon deletion, the GeneTrap mice were crossed with FLPe mice (Dymecki, 1996) to generate a floxed allele (*Iir* ^fl^). *Iir* ^fl/fl^ mice were crossed with Rosa26R-Cre mice (Soriano, 1999) to generate heterozygous *Iir* ^+/−^ mice. *Iir* ^+/−^ mice were backcrossed to a C57BL/6 J (Charles River) background. Genotyping has been performed with the following primers: 5′-CCA​AGG​CCA​GCG​ATA​CAA​CC-3′, 5′-GGA​ACT​TCG​TCG​AGA​TAA​CTT​CGT​ATA​G-3′, 5′-GTG​CAC​TCT​GGG​TAG​TGT​TC-3’. Tissues of males between 9–12 weeks of age were used.

### 2.2 Caudal sperm isolation

The epididymis was isolated, weighed, and cut between the corpus and cauda. The cauda was cut open with several incisions and incubated in HEPES-balanced salt solution (114 mM NaCl, 4.7 mM KCl, 1.2 mM KH_2_PO_4_, 1.16 mM MgSO_4_, 2.5 mM CaCl_2_, 25.5 mM NaHCO_3_, 20 mM HEPES pH 7.2) for 15 min at 37°C. A sperm sample from this suspension was heat-inactivated for 2 minutes at 60°C and loaded on a hemocytometer to calculate the total sperm count per cauda epididymis.

### 2.3 Sperm motility assay

Isolated caudal sperm was assessed for motility in pre-warmed M2 medium (Sigma-Aldrich) using a Zeiss Axio Vert. A1 microscope with a 20 × objective. Five different fields of approximately 5 s were recorded by the STC-MC152USB camera (Sentech) in the XYClone software (Hamilton Thorne, V5.12.0.32243) and assessed for moving sperm (motility) and progressing sperm (progressive motility). A minimum of 130 cells were evaluated for each animal.

### 2.4 Immunofluorescence

Isolated testes were fixed in 4% paraformaldehyde (PFA) at 4°C overnight. The fixed tissue was dehydrated in sucrose (10% and 30%) and embedded in tissue freezing medium (Leica). Sections of 10 µm thickness were cut at −20°C, dried at room temperature, and kept frozen at −20°C. Frozen sections were thawed at room temperature, washed with PBS, and permeabilized with 0.2% Triton-X100, followed by blocking with 3% donkey serum, 10% fetal calf serum (FCS), and 0.1% bovine serum albumin (BSA) in PBS-T. Primary antibodies were incubated at 4°C overnight (anti-TGN38, 1:50, NBP1-03495SS, Novus Biologicals; anti-GATA-4, 1:200, 14–9980–80, Thermo Fisher Scientific; anti-DDX4, 1:200, 8761S, Cell Signaling Technology; anti-LAMP2, 1:200, ab13524, anti-CI-M6PR, 1:200, PA3-850, ThermoFisher Scientific; Abcam; anti-inceptor, 1:250, 2G6 (Ansarullah et al., 2021)). Secondary antibodies were used at 1:600 with 4’,6-diamidino-2-phenylindole (DAPI) at 1 μg/mL for 2 hours at room temperature (anti-rat-Alexa Fluor™ 488, A-21208, Thermo Fisher Scientific; anti-rat-Cy3, 712–165–153, Jackson ImmunoResearch; anti-rabbit-Alexa Fluor™ 488, A21206, Thermo Fisher Scientific). Peanut agglutinin (PNA)-CY5 (CL-1075–1, Vector Laboratories) was used at 1:1000 added to the secondary antibody mix. The slides were mounted with coverslips in Elvanol and imaged on Zeiss LSM 880 with or without Airyscan Fast mode with a 20 × or 63 × objective. Images were analyzed by Fiji (Schindelin et al., 2012) and treated equally within one experiment, unless stated otherwise, by adjusting brightness, and contrast, and for Figures 2B, D, E, noise was reduced by removing outliers with a two-pixel radius. Maximum intensity projection is shown when stated in the respective figure legend.

For mitochondria staining in caudal sperm, live sperm suspensions were incubated with 100 nM MitoTracker™ Red FM (Invitrogen) and 2.5 ng/mL Hoechst 33342 dye (Invitrogen) for 15 min at 37°C. Samples were centrifuged for 5 min at 600 g and the pellet was resuspended in 100 µL pre-warmed M2 media. Epifluorescence images were taken with a Keyence BZ-9000E microscope with a 20 × objective and captured in the BZ-II Viewer software (Keyence). The channels were merged in Fiji.

### 2.5 Transmission electron microscopy (TEM)

Isolated murine testes were fixed in 4% PFA in 100 mM phosphate buffer pH 7.4 at room temperature for 30 min, then cut in half and fixed for another 1.5 h. For embedding into epoxy resin, the fixed tissue was kept in 4% PFA, for Tokuyasu cryo-sectioning, the tissue was transferred to 1% PFA until further processing. For epoxy resin embedding, the samples were processed according to a modified protocol using osmium tetroxide (OsO_4_), thiocarbohydrazide (TCH), and again OsO_4_ to generate enhanced membrane contrast (Hanker et al., 1966; Deerinck et al., 2010; Völkner et al., 2022). In brief, samples were postfixed overnight in modified Karnovsky fixative (2% glutaraldehyde/2% formaldehyde in 50 mM HEPES, pH 7.4), followed by post-fixation in a 2% aqueous OsO_4_ solution containing 1.5% potassium ferrocyanide and 2 mM CaCl_2_ (30 min on ice), washes in water, 1% TCH in water (20 min at room temperature), washes in water and a second osmium contrasting step in 2% OsO_4_/water (30 min on ice). Samples were washed in water and *en-bloc* contrasted with 1% uranyl acetate/water for 2 h on ice, washed again in water, and dehydrated in a graded series of ethanol/water mixtures (30%, 50%, 70%, 90%, 96%), followed by three changes in pure ethanol on molecular sieve. Samples were infiltrated into EMBed 812 (resin/ethanol mixtures: 1:3, 1:1, 3:1 for 1 h each, followed by pure resin overnight and for 5 h), embedded in flat embedding molds, and cured at 65°C overnight. Ultrathin sections (70 nm) were prepared with a Leica UC6 ultramicrotome (Leica Microsystems) using a diamond knife (Diatome), collected on formvar-coated slot grids, and stained with lead citrate (Venable and Coggeshall, 1965) and uranyl acetate. For Tokuyasu-cryosectioning and immunogold labeling, the samples were processed as described previously (Tokuyasu, 1980; Slot and Geuze, 2007; Völkner et al., 2022). First, they were washed in phosphate buffer, infiltrated stepwise into 10% gelatin (1% for 30 min, 3% for 45 min, 7% for 1 h, 10% for 2 h) at 37°C, cooled down on ice, cut into small blocks, incubated in 2.3 M sucrose/water for 24 h at 4°C, mounted on pins (Leica # 16701950), and plunge frozen in liquid nitrogen. 70–100 nm sections were cut on a Leica UC6+FC6 cryo-ultramicrotome (Leica Microsystems) and picked up in methyl cellulose/sucrose (1 part 2% methyl cellulose (MC, Sigma M-6385, 25 centipoises +1 part 2.3 M sucrose) using a perfect loop. Gelatin, sucrose, and methyl cellulose were removed by placing the grids on 37°C warm PBS for 3 × 20 min, followed by washes with 0.1% glycin/PBS (5 × 1 min), blocking with 1% BSA/PBS (2 × 5 min) and incubation with the primary antibody (rat anti-inceptor 16F6 (Ansarullah et al., 2021), 1:100) for 1 h. The sections were washed in PBS (4 × 2 min) and incubated with bridging antibodies (rabbit-anti-mouse or rabbit-anti-rat IgGs, 1:100), followed by washes in PBS (4 × 2 min), and incubation with protein A conjugated to 10 nm gold (1:25, UMC) for 1 h. Then, the grids were washed in PBS (3 × 5 s, 4 × 2 min), post-fixed in 1% glutaraldehyde (5 min), thoroughly washed in water to get rid of the PBS (10 × 1 min) and contrasted with neutral uranyl oxalate (2% uranyl acetate (UA) in 0.15 M oxalic acid, pH 7.0) for 5 min, washed in water and incubated in MC containing 0.4% UA for 5 min. Grids were looped out with a perfect loop, the MC/UA film was reduced to an even thin film and air dried. All sections were analyzed on a JEM 1400PLus transmission electron microscope (JEOL) at 80 kV and images were taken with a Ruby digital camera (JEOL).

### 2.6 Co-immunoprecipitation (co-IP) and western blotting

Isolated murine testes (n = 6) were homogenized in a Potter-Elvehjem homogenizer in 125 mM KCl, 10 mM EDTA, 20 mM HEPES (pH 7.2), 1% protease inhibitor cocktail (Sigma). The protein concentration was measured by BCA Protein Assay Kit (Thermo Scientific Pierce). Western blot samples were prepared by adding Laemmli buffer and boiling. For co-IP, the homogenate was centrifuged at 2000 *g* for 10 min. To the supernatant, 1% NP-40 alternative (Merck Millipore) was added. For mass spectrometry, the lysate was added to anti-Inceptor antibodies or isotype control antibodies (2G6 and 11A7, respectively (Ansarullah et al., 2021)) coupled to protein G SureBeads (BioRad). For co-IP followed by Western blotting, the following antibodies were coupled to protein G beads: anti-AP1M1 (PA5104319, Thermo Fisher Scientific), anti-AP2B1 (ab205014, Abcam), anti-AP3D1 (anti-delta SA4, DSHB), rabbit control (3900, Cell Signaling Technology), mouse control (5415, Cell Signaling Technology), anti-STX7 (12322-1-AP, Proteintech), anti-LYZL4 (17443-1-AP, Proteintech), anti-MAP1B (ab224115, Abcam), anti-Cathepsin Z (ab182575, Abcam). The co-immunoprecipitated protein was eluted into Laemmli buffer at 99°C for Western blotting or at 60°C for mass spectrometry. The samples were loaded on a 10% or 4%–20% gradient SDS-polyacrylamide gel. The separated protein was transferred to a PVDF membrane with 0.2 μm pore-size by semi-dry transfer (BioRad). Blotted membranes were blocked in 5% milk in TBS-T, incubated with primary antibodies at 4°C overnight (anti-SPACA1, 1:1000, ab191843, Abcam; anti-γ-tubulin, 1:2000, T5326, Sigma-Aldrich; anti-β-adaptin, 1:1000, 610382, BD Biosciences; anti-GAPDH, 1:4000, 2118L, Cell Signaling Technology; anti-inceptor, 1:1000, 16F6 (Ansarullah et al., 2021); anti-LYZL4, 1:1000, 17443-1-AP, Proteintech; anti-HSP90, 1:5000, 4874S, Cell Signaling Technology) and with secondary antibodies 1:5000 for 1 h at room temperature (anti-rat-HRP, 112-035-175, Jackson ImmunoResearch; anti-mouse-HRP, 115-035-146, Jackson ImmunoResearch; anti-rabbit-HRP, 111-035-144, Jackson ImmunoResearch). The chemiluminescence signal was detected by ChemStudio2A (Analytik Jena) using Clarity Western ECL Substrate (Bio-Rad).

### 2.7 Mass spectrometry

For total proteome, 1% NP-40 alternative (Merck Millipore) was added to homogenized testis, prepared as described above. For the interactome analysis, the eluate from the co-IP described above was used. The samples were further proteolyzed with LysC and trypsin as described (Wiśniewski et al., 2009; Grosche et al., 2016). Acidified eluted peptides were analyzed on a Q Exactive HF-X mass spectrometer (Thermo Fisher Scientific) online coupled to a UItimate 3000 RSLC nano-HPLC (Dionex) on a C18 analytical column, separated by a 90-min non-linear acetonitrile gradient at a flow rate of 250 nL/min. MS spectra were recorded at a resolution of 60000 with an AGC target of 3 x 1e6 and a maximum injection time of 30 ms from 300 to 1500 m/z. From the MS scan, the 15 most abundant peptide ions were selected for fragmentation via HCD with a normalized collision energy of 28, an isolation window of 1.6 m/z, and a dynamic exclusion of 30 s. MS/MS spectra were recorded at a resolution of 15000 with a AGC target of 1e5 and a maximum injection time of 50 ms. Unassigned charges, and charges of +1 and >8 were excluded from precursor selection.

Acquired raw data of the total testis proteome samples was analyzed in the Proteome Discoverer 2.4 SP1 software (Thermo Fisher Scientific; version 2.4.1.15) for peptide and protein identification via a database search (Sequest HT search engine) against the SwissProt Mouse database (Release 2020_02, 17061 sequences), considering full tryptic specificity, allowing for up to one missed tryptic cleavage site. The Percolator algorithm (Käll et al., 2007) was used for validating peptide spectrum matches and peptides. The final list of proteins satisfying the strict parsimony principle included only protein groups passing an additional protein confidence false discovery rate <5% (target/decoy concatenated search validation). Protein groups with ≥50% missing values were disregarded. In the remaining protein groups, missing values were imputed by GMSimpute (Li et al., 2020). The *p* values were adjusted according to Hochberg (Hochberg, 1988), and we deemed proteins with an adjusted *p* value <0.05 significant. Significant hits were analyzed for gene ontology (GO) term enrichment via the g:Profiler tool g:GOSt (Raudvere et al., 2019) against the custom reference gene list containing all detected proteins after imputation and filtration in the total proteome sample.

Raw data for the interactome analysis was imported into Progenesis QI software (version 4.1). After feature alignment and normalization, spectra were exported as Mascot Generic files and searched against SwissProt Mouse database (Release 2020_02, 17061 sequences) with Mascot (Matrix Science, version 2.8.2) with the following search parameters: 10 ppm peptide mass tolerance and 20 mmu fragment mass tolerance, one missed cleavage allowed, carbamidomethylation was set as fixed modification, methionine oxidation, and asparagine or glutamine deamidation were allowed as variable modifications. A Mascot-integrated decoy database search calculated an average false discovery rate <0.5% for PSMs when searches were performed applying the mascot percolator score and a significance threshold *p* < 0.05. Peptide assignments were re-imported into the Progenesis QI software and the abundances of all unique peptides allocated to each protein were summed up. The resulting normalized abundances of the individual proteins were used for calculation of fold-changes of protein ratios between experimental conditions and statistical analysis was performed on log2 transformed normalized abundance values using one-way ANOVA. Values are corrected for multiple testing by an optimized FDR approach (*q* value). Potential interactors with a *q* value <0.05, a fold change between inceptor and control IP > 2, and less than seven missing values (corresponding to 60%) were selected and a GO:term analysis was performed. GO:BP terms were reduced with the rrvgo R package 1.10.0 (Sayols, 2020) with a threshold of 0.6. The mass spectrometry proteomics data have been deposited to the ProteomeXchange Consortium via the PRIDE (Perez-Riverol et al., 2022) partner repository with the dataset identifier PXD043946.

### 2.8 *Iir* expression across cell types

Normalized transcripts per million (nTPM) data from scRNA-seq datasets were downloaded from The Human Protein Atlas and the 20 cell types with nTPM >20 were plotted for *Elapor1* expression. Cell type group information was manually added.

### 2.9 Statistical analysis and software

Statistical analysis and the generation of bar plots were performed in GraphPad Prism 9.5.1, using unpaired *t*-test. Bar plots are shown as mean ± SD. Mass spectrometry raw data were analyzed in Proteome Discoverer 2.4 SP1 (Thermo Fisher Scientific; version 2.4.1.15) and Progenesis QI software (version 4.1). GO term annotation was performed by g:Profiler (Raudvere et al., 2019) and GO:BP term reduction was done by the rrvgo R package 1.10.0 (Sayols, 2020). The volcano plot and GO term bar plots were generated in RStudio (2022.12.0 + 353, R version 4.2.2) by ggplot2. Image acquisition was done by ZEN 2.3 (black edition, Zeiss), XYClone (Hamilton Thorne), and BZ-II Viewer (Keyence) and image processing was done in Fiji (ImageJ 1.53o) (Schindelin et al., 2012). Figures were generated in Inkscape 1.2.1.

## 3 Results

Inceptor is highly expressed in several tissues, such as the digestive tract, pancreas, prostate, female reproductive tract, lung, brain, and testis, specifically enriched in glandular cell types, endocrine cells, and early spermatids (Supplementary Figure S1A) (The Human Protein Atlas). We confirmed inceptor protein expression in the stomach, salivary gland, colon, and lung by immunofluorescence (Supplementary Figure S1B).

**FIGURE 1:**
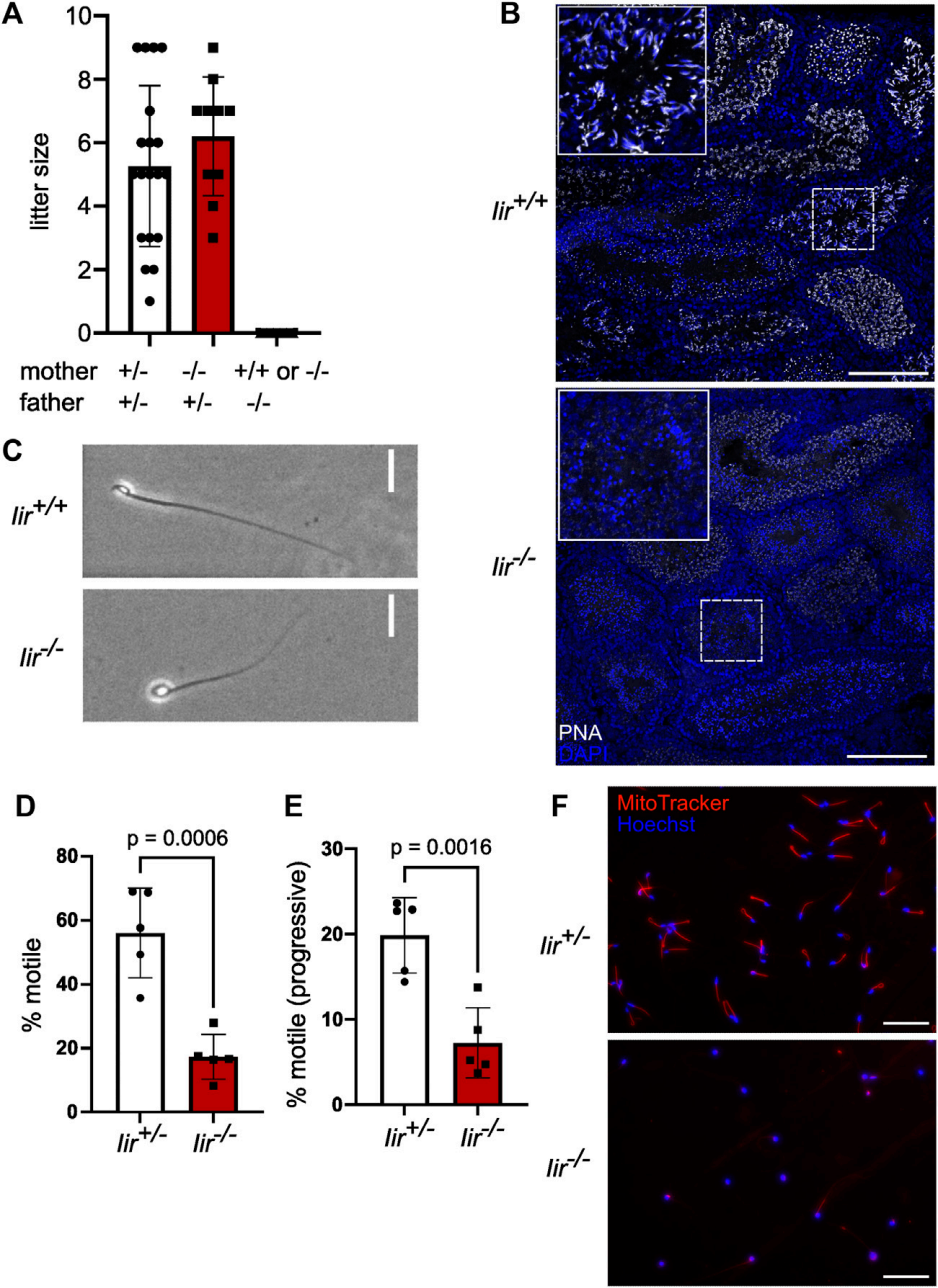
*Iir* ^*−/−*^ male mice are infertile and *Iir* ^*−/−*^ spermatids show acrosome malformation and a globular shape. **(A)** Litter size of crossing *Iir* ^*+/−*^ and *Iir* ^*−/−*^ mice. **(B)** Representative PNA marking acrosomes and DAPI staining marking nuclei in testis sections of *Iir* ^*+/+*^ and *Iir* ^*−/−*^ mice. Maximum-intensity projection. Scale bar 150 μm. **(C)** Representative phase-contrast image of isolated caudal sperm. Scale bar 20 μm. **(D–E)** percentage of motile **(D)** and progressive-motile **(E)** isolated caudal sperm. Each dot represents sperm isolated from one mouse consisting of five measurements per mouse **(F)** Representative MitoTracker staining with Hoechst 33342 counterstain of isolated caudal sperm from *Iir* ^*+/−*^ and *Iir* ^*−/−*^ mice. Scale bar 100 μm.

To analyze the function of inceptor, we generated the *Iir* ^*+/−*^ mouse line by crossing the previously generated GeneTrap line (Ansarullah et al., 2021) to FLPe and RosaCre animals to obtain a germline deletion of exon 3 (Supplementary Figure S1C). By heterozygous intercrossing of the *Iir* ^*+/−*^ mouse line, Mendelian ratios of offspring genotypes were observed at weaning age (Supplementary Figure S1D). We confirmed the absence of inceptor in *Iir* ^*−/−*^ testis lysates by Western blotting (Supplementary Figure S1E). Intriguingly, the *Iir* ^*−/−*^ genotype could not be maintained on a homozygous background. While *Iir* ^*−/−*^ females delivered similar litter sizes as *Iir* ^*+/−*^ females with *Iir* ^*+/−*^ males, matings with *Iir* ^*−/−*^ males were unsuccessful (Figure 1A). These results are consistent with previously described infertility of *Iir* ^*−/−*^ males (Tang et al., 2010; Cho et al., 2022), however, the underlying cellular and molecular mechanism was unknown.

To further investigate the function of inceptor in male fertility, we analyzed testis sections in *Iir* ^*+/+*^ and *Iir* ^*−/−*^ mice. Interestingly, we noticed partially or totally missing PNA staining, indicating failure in acrosome formation (Figure 1B). The round nuclei of *Iir* ^*−/−*^ spermatozoa are indicative of a condition referred to as globozoospermia (Figure 1C). Globozoospermia and acrosome malformation are often accompanied by motility and mitochondria defects (Xiao et al., 2009; Han et al., 2017). Indeed, *Iir* ^*−/−*^ spermatozoa are characterized by reduced motility, as well as defective mitochondrial organization (Figures 1D–F).

*Iir* ^*+/+*^ and in *Iir* ^*−/−*^ males did not differ in body weight (Supplementary Figure S2A), while *Iir* ^*−/−*^ males had a slightly increased testis weight without a significant difference in caudal sperm count (Supplementary Figures S2B, C). There was no difference in gross morphology of the testis and epididymis (Supplementary Figure S2D). By analyzing testicular cross sections by immunofluorescence, there were no differences between *Iir* ^*+/+*^ and *Iir* ^*−/−*^ testes in germ cells marked by DDX4 and Sertoli cells marked by GATA-4 (Supplementary Figure S2E), as well as tubule diameter (Supplementary Figure S2F).

**FIGURE 2:**
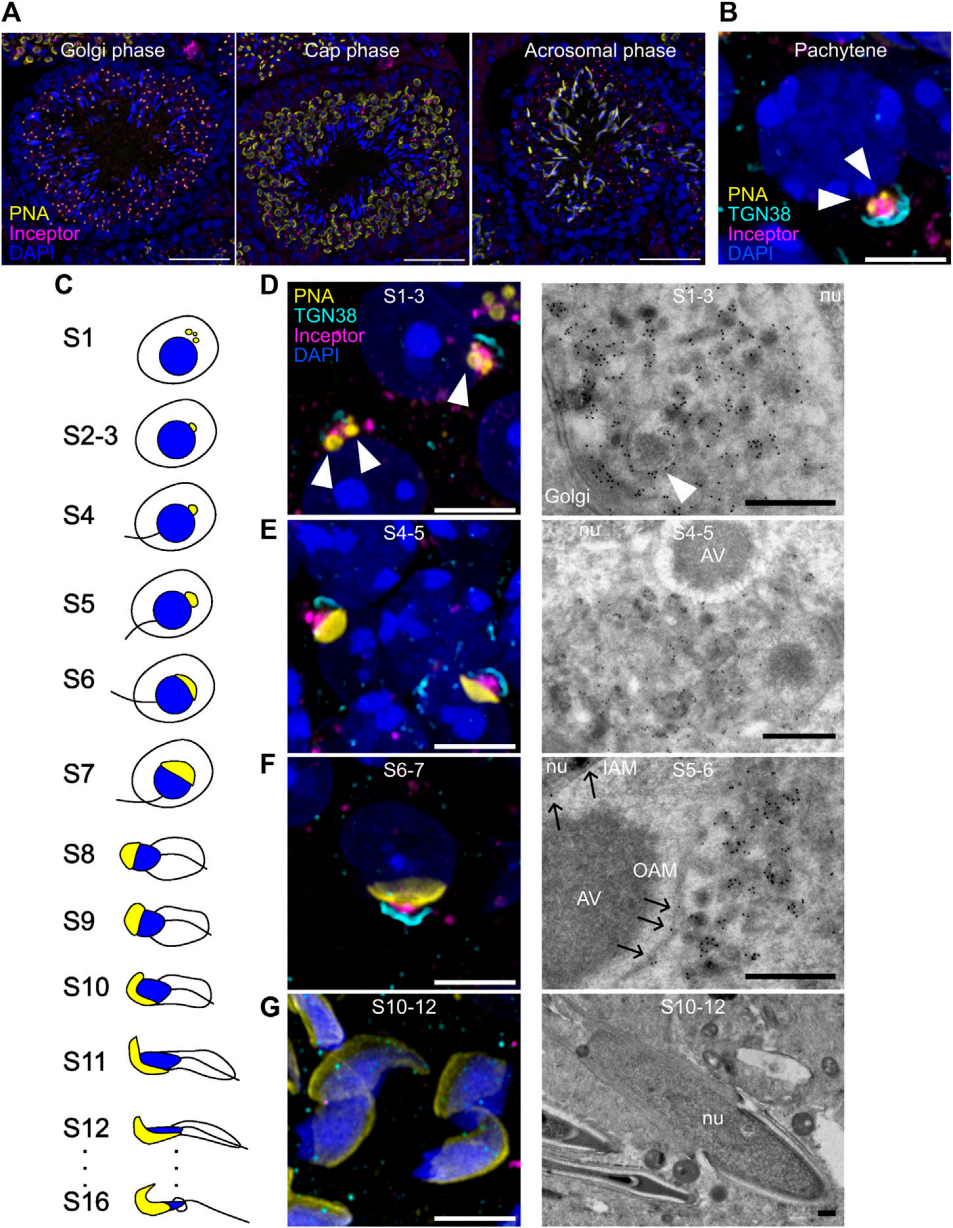
Inceptor localizes to the pro-AVs and acrosome in early-stage spermatids. **(A)** Representative immunofluorescence images of inceptor co-stained with PNA and DAPI, maximum intensity projection. Scale bar 50 μm. **(B)** Representative immunofluorescence images of inceptor and TGN38 co-stained with PNA and DAPI in the pachytene stage. **(C)** Schematic overview of spermatid development **(D–G)** Representative immunofluorescence images for stages S1-3, **(E)** S4-5, **(F)** S6-7, **(G)** S10-12 (left panels) and immunogold labeling of inceptor (right panels). **(B,D–G)** Airyscan acquisition and processing, maximum intensity projection. Scale bar 10 μm for immunofluorescence images, scale bar 2 μm for TEM images. nu, nucleus; AV, acrosomal vesicle; IAM, inner acrosomal membrane; OAM, outer acrosomal membrane. White arrowheads: pro-AVs, black arrows: inceptor localization to the acrosomal membrane.

In the seminiferous tubules of the testis, inceptor is expressed in spermatocytes and in Golgi-phase and cap-phase spermatids but not in elongated spermatids in the acrosomal phase (Figure 2A). By confocal microscopy, we found inceptor in the vesicles of pachytene spermatocytes when the pro-AVs are harbored close to the Golgi apparatus and not yet attached to the nuclear membrane (Figure 2B). After meiosis, spermatids develop in 16 steps into spermatozoa (Oakberg, 1956; Wakayama et al., 2022) (Figure 2C). Confocal microscopy and TEM of immunogold labeled Tokuyasu-cryosections showed that inceptor is localized to pro-AVs and other vesicles before stage 3 (S3), before the pro-AVs attach to the nucleus (Figure 2D). Next, pro-AVs start to fuse into a cap-like structure (up to S7), when inceptor is predominantly present in the vesicles trafficking between the Golgi and the AV, and to a lesser extent in the inner and outer acrosomal membranes (Figures 2E, F). Then, the nucleus starts to elongate while the AV expands to cover most of the nuclear surface. The cell discards the Golgi apparatus and other organelles at this stage, while the inceptor immunosignal is completely lost (Figure 2G). In conclusion, in the early stages of acrosome formation, inceptor is localized to trafficking vesicles, pro-AVs, and the inner and outer acrosomal membranes of the developing AV.

**FIGURE 3:**
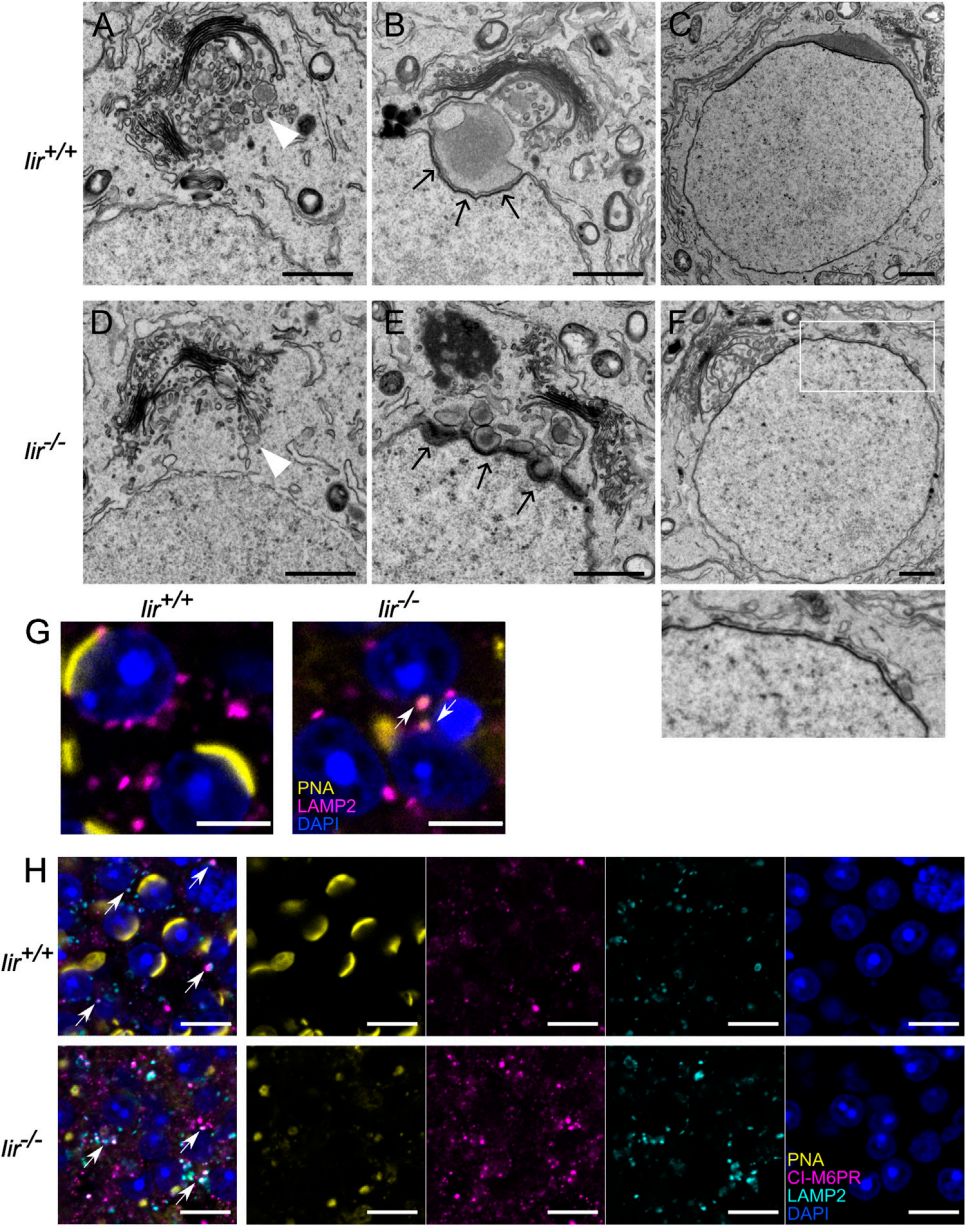
Pro-AVs do not fuse in *Iir* ^*−/−*^ spermatids, resulting in a scrambled acrosome **(A–F)** Representative electron micrographs of early-stage spermatids. Scale bars 1 μm. White arrowheads: pro-AVs, black arrows: acroplaxome **(G)** Example immunofluorescence image of LAMP2 and PNA overlapping in *Iir* ^*+/+*^ and *Iir* ^*−/−*^ spermatids. Scale bar 5 μm. White arrows point at PNA-positive lysosomes **(H)** Representative immunofluorescence image of CI-M6PR and LAMP2-positive vesicle localization in *Iir* ^*+/+*^ and *Iir* ^*−/−*^ spermatids and PNA and DAPI counterstain. Scale bar 10 μm. White arrows point at LAMP2 and CI-M6PR colocalization.

As inceptor expression coincides with the early steps of acrosome formation, we used TEM to visualize the acrosomal shape in *Iir* ^*+/+*^ and *Iir* ^*−/−*^ mice. As expected, in *Iir* ^*+/+*^ mice, we observed electron-dense pro-AVs before acrosome formation, that are around 200-μm large in diameter (Figure 3A, white arrowhead). During pro-AV fusion, the single AV attaches to the nucleus at the beginning of the cap phase. At the nuclear membrane and AV contact site, the electron-dense acroplaxome is visible (Figure 3B, black arrows). As the AV expands and covers more nuclear surface, the acroplaxome also grows along the contact site (Figure 3C). After that, the nucleus and AV elongate, and the nucleus becomes more electron-dense (Supplementary Figures S3A–C). In *Iir* ^*−/−*^ testis, pro-AVs localize to the *trans*-Golgi network as in the *Iir* ^*+/+*^ controls before acrosome formation (Figure 3D). At the beginning of the cap phase, however, pro-AVs do not form one large AV but remain segregated into around 200-μm large vesicles (Figure 3E). These vesicles are in close proximity to the nucleus, suggesting the correct delivery but failure to fuse at the acroplaxome. In later stages, the electron-dense acroplaxome expands around the nucleus, but the AV consists of small, irregularly distributed electron-dense vesicles in *Iir* ^*−/−*^ mice (Figure 3F). In further stages of acrosome formation, the nucleus fails to elongate and remains round or irregular in shape (Supplementary Figures S3D–F). By immunofluorescence, we observed some of the scattered acrosomal content marked by PNA localized to LAMP2-positive lysosomes, whereas in *Iir* ^*+/+*^ cap-phase spermatids, LAMP2-positive lysosomes mainly localized to the posterior side of the spermatid (Figure 3G). Due to the similarity between inceptor and M6PRs, we investigated the localization of the cation-independent (CI-)M6PR and confirmed that CI-M6PR localization resembles a more diffuse vesicular localization than inceptor, with occasional colocalization with LAMP2-positive lysosomes (Figure 3H, white arrows).

**FIGURE 4:**
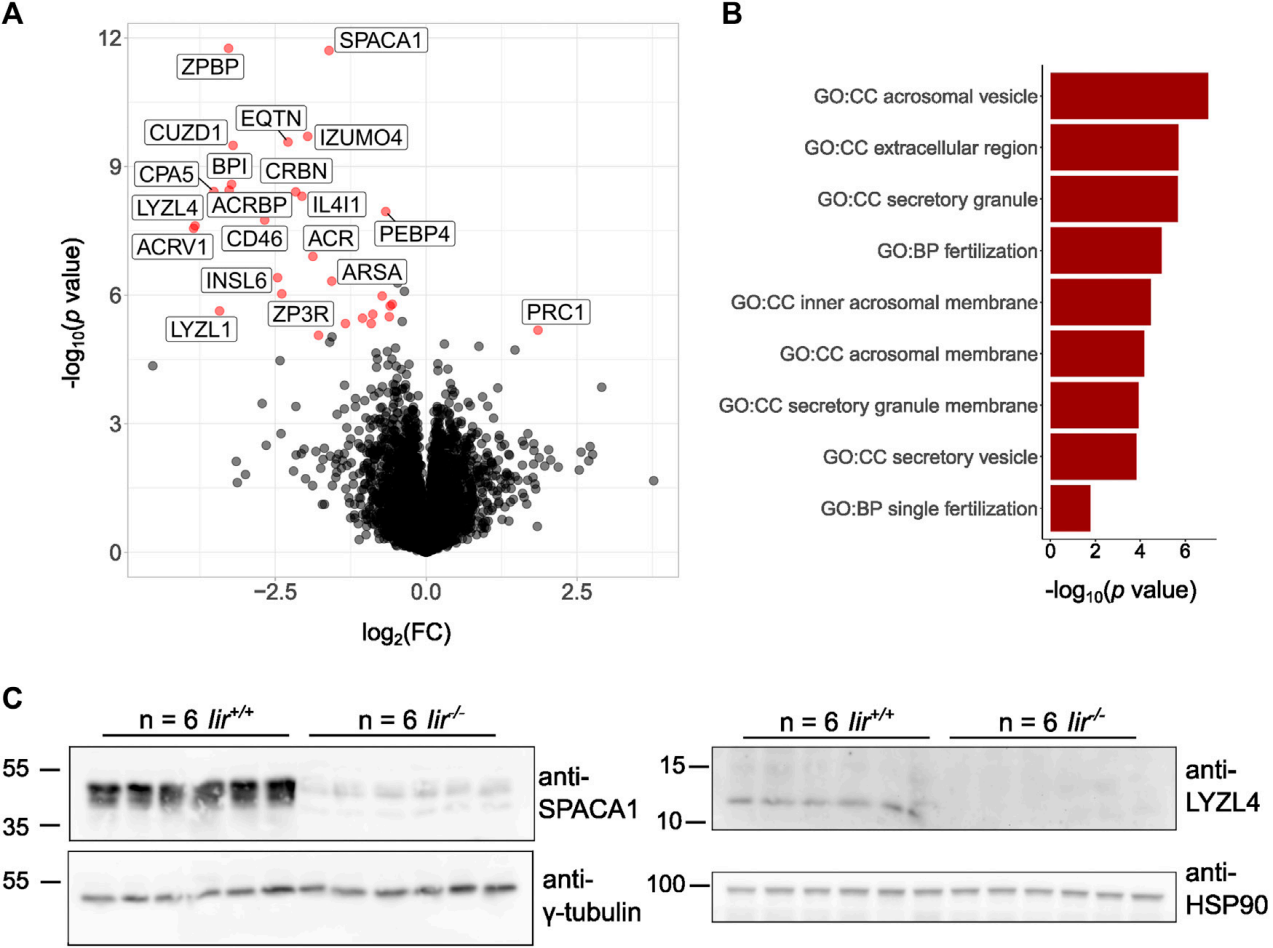
*Iir* ^*−/−*^ testes lack acrosomal proteins essential for fertilization. **(A)** Volcano plot of the total testis proteome of *Iir* ^*−/−*^ vs *Iir* ^*+/+*^ mice. Red dots highlight significant hits. **(B)** Significant GO terms from significant hits from **(A)** against all proteins detected in **(A)**. **(C)** Validation of the downregulation of SPACA1 and LYZL4 by Western blot.

As we observed small, deformed, or lacking acrosomes in *Iir* ^*−/−*^ spermatids, we performed whole-testis proteome analysis on *Iir* ^*+/+*^ and *Iir* ^*−/−*^ testes to provide an unbiased picture of differential regulation of spermatocyte development. For the analysis, we considered 5389 considered protein groups, from which there were 28 significantly downregulated and one significantly upregulated protein (Figure 4A, Supplementary Table S1). We performed a GO term analysis of the significant hits on the background of all 5389 considered protein groups, which shows significantly deregulated terms related to acrosomes, secretion, and fertilization (Figure 4B). Specifically, we see significant downregulation of proteins localized to the acrosome, either soluble or transmembrane proteins. Many well-known proteins essential for fertilization are strongly downregulated in *Iir* ^*−/−*^ testes. Among these proteins were Acrosin (ACR), Acrosin-binding protein (ACRBP), Acrosomal vesicle protein 1 (ACRV1), Sperm acrosome membrane-associated 1 (SPACA1), and Equatorin (EQTN), which are among the main constituents of the acrosome. Additionally, Izumo sperm-egg fusion protein 4 (IZUMO4), and the *Zona-pellucida* binding protein (ZPBP), which have direct functions in sperm-egg fusion, were also downregulated. CD46 and TMEM190 are proteins specifically localized to the inner acrosomal membrane and we also found them downregulated in *Iir* ^*−/−*^ testes. Interestingly, Insulin-like factor 6 (INSL6) is also downregulated, a member of the insulin superfamily expressed specifically in the testis with expression onset in pachytene spermatocytes (Burnicka-Turek et al., 2009). We confirmed the downregulation of SPACA1 and Lysozyme-like 4 (LYZL4) by Western blot (Figure 4C).

**FIGURE 5:**
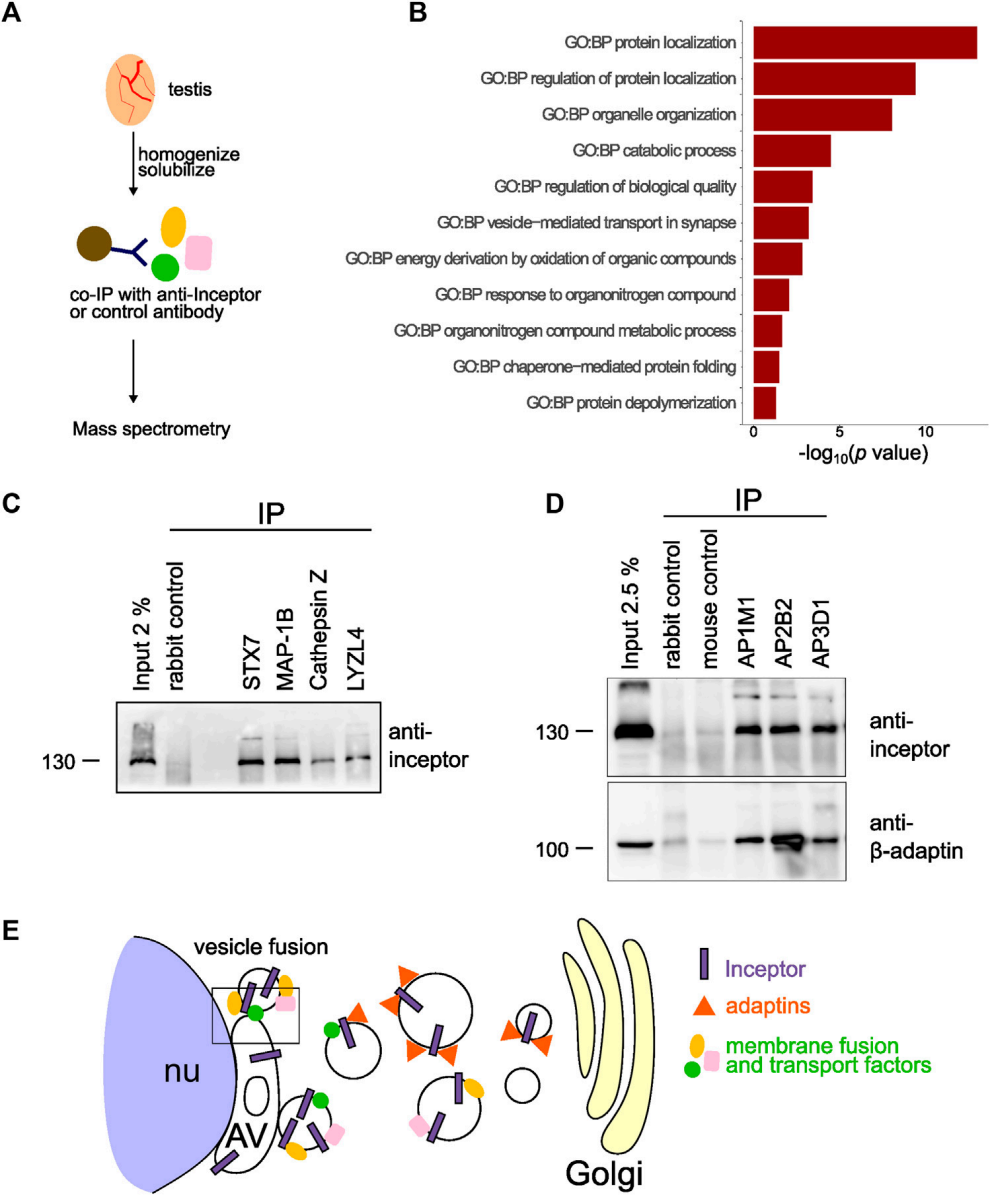
Inceptor mediates subcellular localization and transport in maturing spermatids **(A)** Schematic representation of the experimental setup. **(B)** Parental GO:BP terms of co-immunoprecipitated proteins with inceptor in whole testis. **(C)** Validation of the mass spectrometry results by co-IP of potential interactors. **(D)** Western blot of adaptor protein co-IP for inceptor and *β*-adaptin. **(E)** Schematic overview of inceptor localization and function in early-stage spermatids.

As inceptor localizes between the Golgi and AV and in its absence the acrosome does not fully develop, we hypothesized that inceptor is necessary for trafficking between the Golgi and AV in developing spermatids. Therefore, the interaction partners and potential cargo of inceptor became of interest in deciphering the underlying mechanism of inceptor’s role in acrosome formation. We performed an inceptor co-IP experiment followed by mass spectrometry analysis (Figure 5A). We analyzed GO terms related to biological processes (GO:BP) on the 128 potential interactors (Supplementary Table S2). The significant GO:BP terms were reduced to eleven parental terms (Supplementary Figure S4). This GO:BP enrichment analysis revealed that inceptor interacts with proteins related to protein localization, organelle organization, catabolic process and vesicle-mediated transport (Figure 5B). From the list of candidate interactors, we validated the binding of inceptor to proteins that are well-known for their function in vesicle trafficking and fusion, such as syntaxin 7 (STX7) and microtubule-associated protein 1B (MAP1B) (Figure 5C). We also confirmed the interaction with the significantly downregulated LYZL4 and the M6P-containing cathepsin Z (Figure 5C). We have previously shown inceptor’s YXXθ to bind the *µ* subunit of AP-2 in pancreatic beta cells (Ansarullah et al., 2021), as this motif is known to bind the *µ* subunits of AP1-4 (Braulke and Bonifacino, 2009). Interestingly, we found the adaptor protein 3 μ2 subunit (AP3M2) to interact with inceptor (Supplementary Table S1), so we investigated adaptor protein and inceptor’s interaction in testes. We found that inceptor is bound by the AP complexes 1, 2, and 3, which likely mediate its subcellular localization and trafficking (Figure 5D). In summary, we propose that inceptor mediates vesicle trafficking between the Golgi and AV compartments and facilitates the transport and function of various proteins involved in cellular trafficking, such as proteins involved in vesicle fusion (Figure 5E).

## 4 Discussion

Around seven percent of the human male population has a form of infertility but the underlying disease mechanism remains unknown in most cases (Krausz and Riera-Escamilla, 2018). Moreover, discovering factors that could serve as targets for on-demand male contraception is sought after (Kent et al., 2020). For this purpose, factors essential for spermatogenesis specifically expressed in spermatocytes and spermatids but not in spermatogonia are promising candidates.

Here, we described inceptor expression, localization, and function in the murine testis. Inceptor is essential for the development of morphologically intact, motile, and fertile spermatozoa. Inceptor’s expression onset is in primary spermatocytes, which arise from germ cells by meiosis. The expression is retained until the elongation stage of maturing spermatids. By immunofluorescence and immunogold labeling, we found that inceptor localizes to vesicles shuttling between the Golgi and acrosome compartments, and in its absence, pro-AVs do not fuse to form the acrosome. Together with the downregulation of proteins localized to the acrosome in *Iir* ^*−/−*^ testes and the enrichment of transport and localization-related proteins in inceptor’s interactome, these results indicate, that inceptor mediates intracellular transport to ensure pro-AV fusion. Indeed, we confirm inceptor to bind membrane-fusion and vesicle-trafficking factors, such as STX7 and MAP1B. As inceptor likely does not contain catalytically active domains and we described domains important for interacting with other proteins (Ansarullah et al., 2021), we propose that inceptor does not mediate the membrane fusion of the pro-AVs directly, but has a function in delivering factors that aid the membrane fusion and cargo delivery to the acrosome.

Interestingly, *Pick1* ^*−/−*^ and *Gopc* ^*−/−*^ mice also show defects in acrosome formation and a failure of pro-AV fusion, similarly to *Iir* ^*−/−*^ mice (Yao et al., 2002; Xiao et al., 2009). From these published immunofluorescence images, PICK1 and GOPC seem to localize more broadly in spermatids compared to inceptor and the TEM images suggest that inceptor and PICK1 could be present in the same vesicles. Other mouse KOs also show globozoospermia and defects in acrosome formation, albeit with some differences. For example, *Gm130* ^*−/−*^ spermatids form an AV which then fails to grow, whereas *Dpy19l2* ^*−/−*^ spermatids show a destabilized nuclear membrane (Pierre et al., 2012; Han et al., 2017). *Spaca1* ^*−/−*^ spermatids have a destabilized AV and lack the acroplaxome, whereas *Zpbp* ^*−/−*^ mice have a fragmented acrosome due to failure in acrosome compaction (Lin et al., 2007; Fujihara et al., 2012). These functional studies suggest, that each step of acrosome formation is tightly regulated by a variety of proteins. However, these regulatory steps seem to interconnected, as *Gopc* ^*−/−*^ mice also show reduced ZPBP1 and SPACA1 proteins, and *Zpbp* ^*−/−*^ mice also show a reduction in SPACA1 (Fujihara et al., 2012). Interestingly, neither PICK1, GOPC, nor DPY19L2 levels were changed in *Iir* ^*−/−*^ testes, whereas SPACA1 and ZPBP1 were among the most significantly downregulated proteins compared to *Iir* ^*+/+*^ testes (Supplementary Table S1).

Inceptor binds insulin and proinsulin in pancreatic beta cells and regulates their degradation (Siehler et al., unpublished results). Intriguingly, INSL6, a relaxin-like protein and one of insulin-like factors specifically expressed in testes, was also downregulated in testes of *Iir* ^*−/−*^ mice. INSL6 levels have been found to correlate positively with male fertility in humans (Ivell and Grutzner, 2009; Chen et al., 2011; Ivell et al., 2017; Gumus et al., 2022). Inceptor also shares similarities to CI-M6PR, which transports IGF2 towards lysosomes, as well as acts as transport receptors for hydrolases toward late endosomes and lysosomes. As the acrosome shares similarities in origin and content to lysosomes, one hypothesis could be that M6PRs also contribute towards regulating the acrosomal content. Interestingly, the CI- and cation-dependent (CD)-M6PR have only been found to associate with LAMP1-positive lysosomes and not with acrosin-containing vesicles or acrosomes in spermatids (Martínez-Menárguez et al., 1996), similarly to our results (Figure 3H). In later stages, CI-M6PR has been found to transiently co-localize with acrosomes and has been proposed to contribute to shaping the acrosome (Moreno, 2003). However, in CI- and CD-M6PR mutants, the acrosomal content of selected hydrolases was not affected (Chayko and Orgebin-Crist, 2000). We have confirmed that the M6PR colocalizes with LAMP2-positive lysosomes rather than the acrosome, whereas inceptor is more closely located to the acrosome. In summary, we propose that inceptor is an important lysosomal trafficking receptor in different cell types, and an acrosomal trafficking receptor in developing spermatids.

## Data Availability

The datasets presented in this study can be found in online repositories. The names of the repository/repositories and accession number(s) can be found in the article/Supplementary Material.
