# Supplementary figures and images for: Inceptor facilitates acrosomal vesicle formation in spermatids and is required for male fertility
Source: Front Cell Dev Biol. 2023 Aug 24;11:1240039. doi: 10.3389/fcell.2023.1240039 (PMC10483240; doi:10.3389/fcell.2023.1240039)

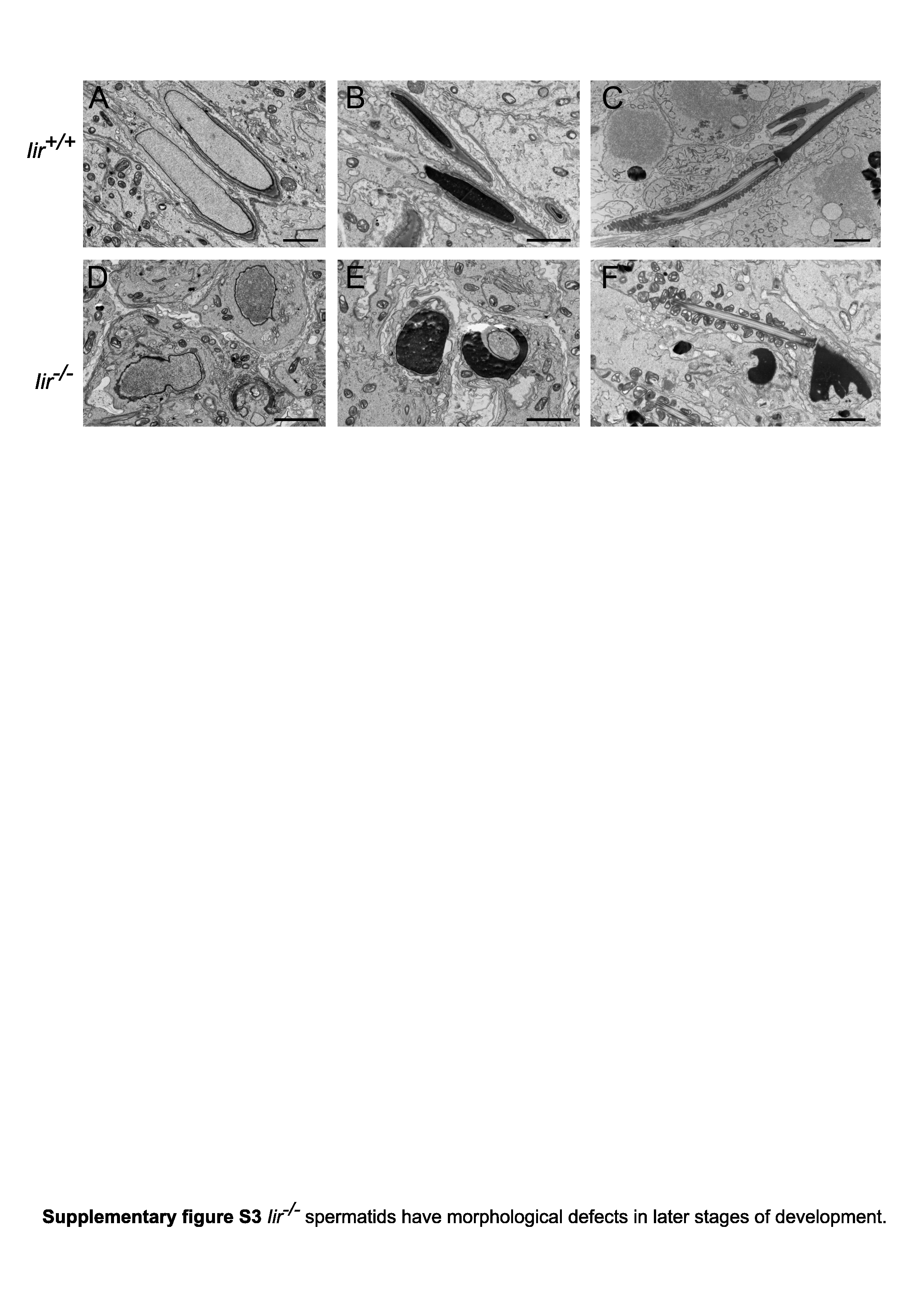

Supplement: Supplementary file 1 [file Image3.jpg]

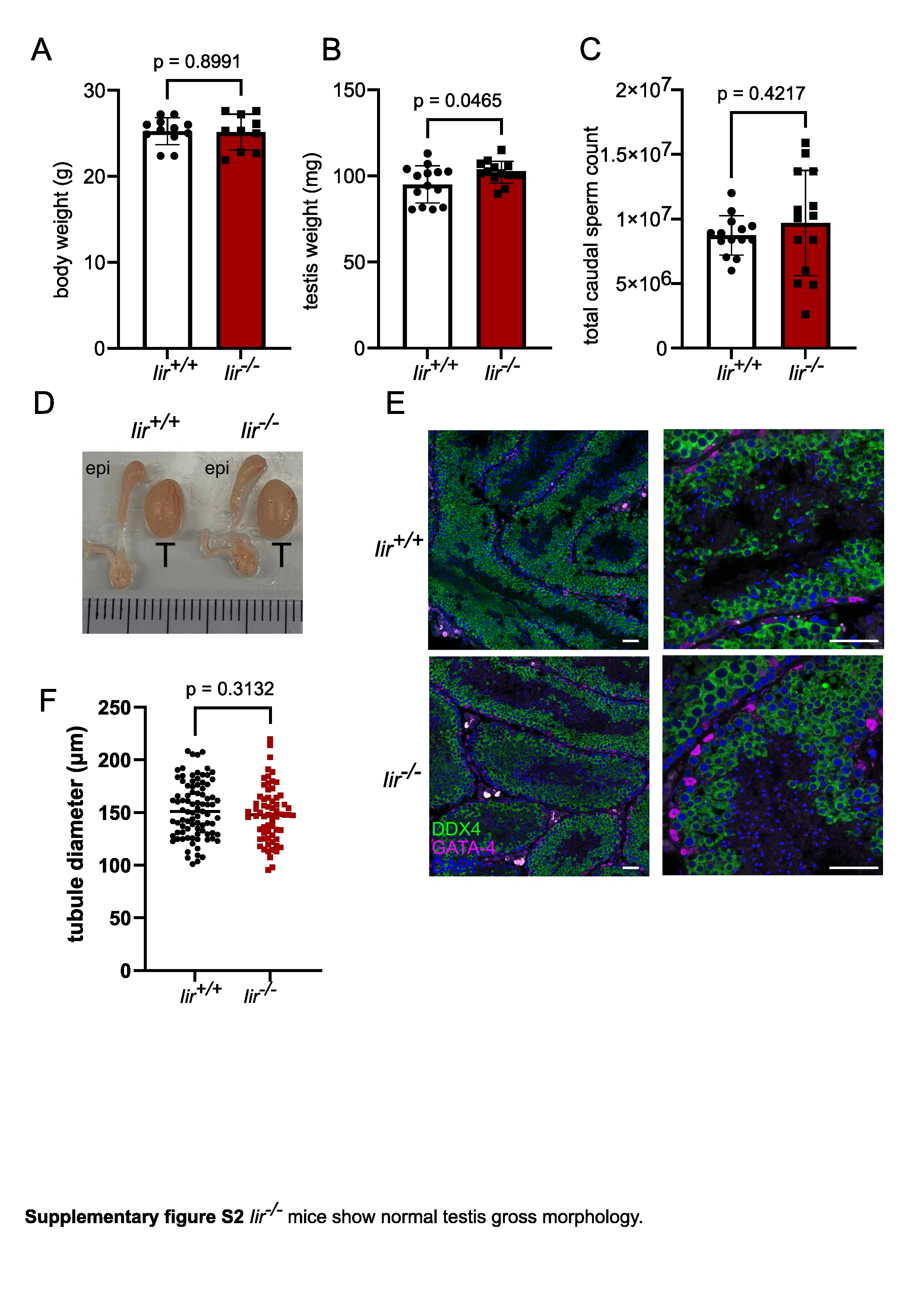

Supplement: Supplementary file 2 [file Image2.jpg]

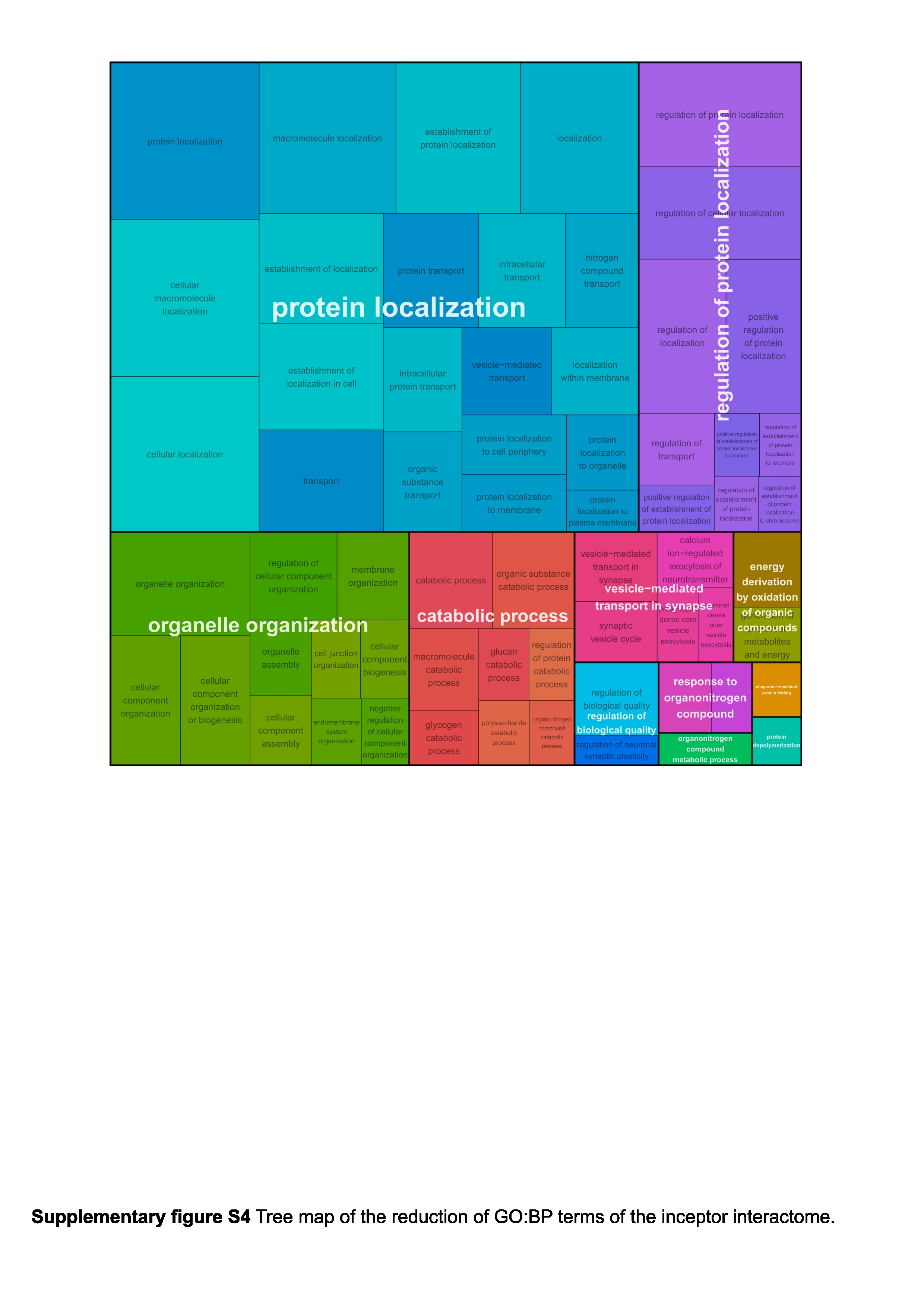

Supplement: Supplementary file 6 [file Image4.jpg]

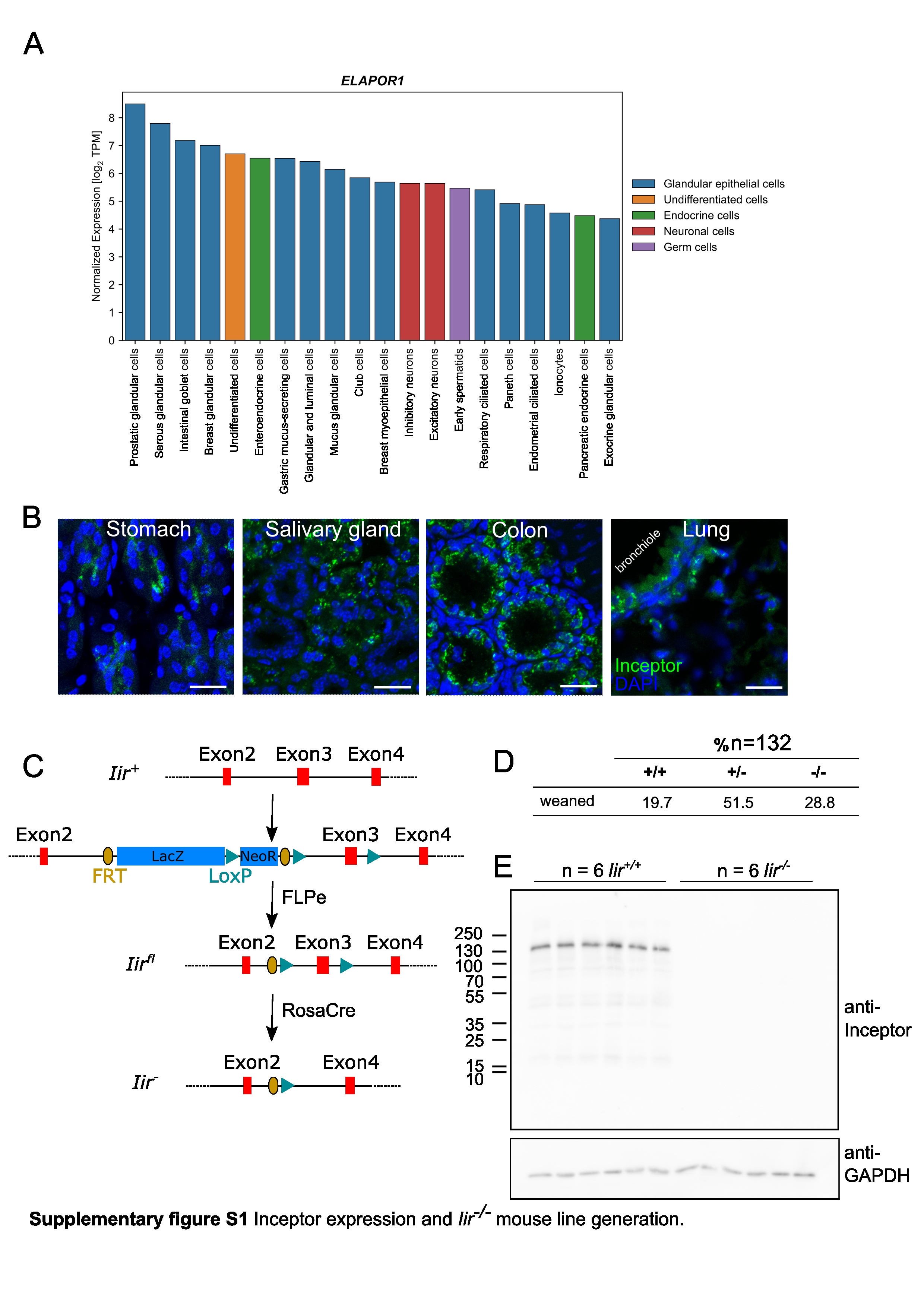

Supplement: Supplementary file 7 [file Image1.jpg]
