# Supplementary material for: Inceptor facilitates acrosomal vesicle formation in spermatids and is required for male fertility
Source: Front Cell Dev Biol. 2023 Aug 24;11:1240039. doi: 10.3389/fcell.2023.1240039 (PMC10483240; doi:10.3389/fcell.2023.1240039)
Supplement: Supplementary file 3 [file Table2.DOCX]

**Supplementary Table 2** - Candidate interactors of inceptor from the mass spectrometry analysis. Arranged from top to bottom by descending fold change compared to a co-IP with a control antibody. The candidates have been filtered for *q* < 0.05, fold change > 2, and < 60 % missing values.

|  | Gene | Description | Fold change | *q* value |
| --- | --- | --- | --- | --- |
| 1 | Nudt21 | Cleavage and polyadenylation specificity factor subunit 5 | Inf | 0.00024 |
| *2* | Ap3m2 | AP-3 complex subunit mu-2 | Inf | 0.00881 |
| *3* | Sh3bgrl | SH3 domain-binding glutamic acid-rich-like protein | Inf | 0.00999 |
| *4* | Gorasp2 | Golgi reassembly-stacking protein 2 | 97159.2 | 0.01328 |
| *5* | Epb41 | Protein 4.1 | 20328.8 | 0.00002 |
| *6* | Pdk1 | [Pyruvate dehydrogenase (acetyl-transferring)] kinase isozyme 1, mitochondrial | 448.3 | 0.00398 |
| *7* | Hephl1 | Ferroxidase HEPHL1 | 405.4 | 0.03284 |
| *8* | Map1a | Microtubule-associated protein 1A | 281.1 | 0.01887 |
| *9* | Ranbp1 | Ran-specific GTPase-activating protein | 250.7 | 0.03524 |
| *10* | Pycr3 | Pyrroline-5-carboxylate reductase 3 | 235.0 | 0.03564 |
| *11* | Snx3 | Sorting nexin-3 | 144.7 | 0.00103 |
| *12* | Dlg4 | Disks large homolog 4 | 141.9 | 0.02217 |
| *13* | Rpe65 | Retinoid isomerohydrolase | 141.8 | 0.04080 |
| *14* | Nucks1 | Nuclear ubiquitous casein and cyclin-dependent kinase substrate 1 | 133.6 | 0.02184 |
| *15* | Tmem190 | Transmembrane protein 190 | 114.7 | 0.00103 |
| *16* | Gpm6b | Neuronal membrane glycoprotein M6-b | 112.4 | 0.00467 |
| *17* | Pdxk | Pyridoxal kinase | 83.3 | 0.00618 |
| *18* | Dctn5 | Dynactin subunit 5 | 76.0 | 0.00233 |
| *19* | Pgm2 | Phosphoglucomutase-2 | 69.9 | 0.00176 |
| *20* | Basp1 | Brain acid soluble protein 1 | 68.6 | 0.02720 |
| *21* | Septin6 | Septin-6 | 55.7 | 0.02944 |
| *22* | Dnajb2 | DnaJ homolog subfamily B member 2 | 51.4 | 0.00854 |
| *23* | Tax1bp1 | Tax1-binding protein 1 homolog | 47.6 | 0.02445 |
| *24* | Msn | Moesin | 41.9 | 0.01004 |
| *25* | Gnb5 | Guanine nucleotide-binding protein subunit beta-5 | 41.0 | 0.01762 |
| *26* | Arhgdia | Rho GDP-dissociation inhibitor 1 | 36.4 | 0.00036 |
| *27* | Atp5md | ATP synthase membrane subunit DAPIT, mitochondrial | 35.2 | 0.02846 |
| *28* | Eprs1 | Bifunctional glutamate/proline--tRNA ligase | 32.7 | 0.04879 |
| *29* | Pcmt1 | Protein-L-isoaspartate(D-aspartate) O-methyltransferase | 32.7 | 0.02360 |
| *30* | Pgm1 | Phosphoglucomutase-1 | 32.5 | 0.00000 |
| *31* | Nrbp1 | Nuclear receptor-binding protein | 32.1 | 0.01056 |
| *32* | Syngr1 | Synaptogyrin-1 | 31.5 | 0.00704 |
| *33* | Syn1 | Synapsin-1 | 30.9 | 0.00803 |
| *34* | Stx7 | Syntaxin-7 | 30.6 | 0.00557 |
| *35* | Psmc6 | 26S proteasome regulatory subunit 10B | 28.3 | 0.04948 |
| *36* | Pacs1 | Phosphofurin acidic cluster sorting protein 1 | 27.8 | 0.04763 |
| *37* | Kiaa0753 | Protein moonraker | 27.7 | 0.00054 |
| *38* | Pkp3 | Plakophilin-3 | 27.5 | 0.04959 |
| *39* | Pafah1b1 | Platelet-activating factor acetylhydrolase IB subunit alpha | 27.4 | 0.00104 |
| *40* | Dnajb6 | DnaJ homolog subfamily B member 6 | 25.6 | 0.01016 |
| *41* | Arl6 | ADP-ribosylation factor-like protein 6 | 25.2 | 0.04948 |
| *42* | Slc25a11 | Mitochondrial 2-oxoglutarate/malate carrier protein | 24.8 | 0.01371 |
| *43* | Capg | Macrophage-capping protein | 22.0 | 0.02720 |
| *44* | Psmg3 | Proteasome assembly chaperone 3 | 21.4 | 0.04059 |
| *45* | Auh | Methylglutaconyl-CoA hydratase, mitochondrial | 21.2 | 0.03994 |
| *46* | Ncam2 | Neural cell adhesion molecule 2 | 21.0 | 0.03524 |
| *47* | Uqcrfs1 | Cytochrome b-c1 complex subunit Rieske, mitochondrial | 19.6 | 0.02846 |
| *48* | Atp2a2 | Sarcoplasmic/endoplasmic reticulum calcium ATPase 2 | 19.2 | 0.00621 |
| *49* | Dctn1 | Dynactin subunit 1 | 17.6 | 0.02293 |
| *50* | Immt | MICOS complex subunit Mic60 | 17.6 | 0.02918 |
| *51* | Hdgfl3 | Hepatoma-derived growth factor-related protein 3 | 16.7 | 0.02846 |
| *52* | Arf3 | ADP-ribosylation factor 3 | 16.3 | 0.00927 |
| *53* | Snx27 | Sorting nexin-27 | 16.2 | 0.03645 |
| *54* | Scamp1 | Secretory carrier-associated membrane protein 1 | 15.6 | 0.03524 |
| *55* | Otub1 | Ubiquitin thioesterase OTUB1 | 15.0 | 0.02431 |
| *56* | Gucy2e | Retinal guanylyl cyclase 1 | 14.7 | 0.02937 |
| *57* | Hnrnpdl | Heterogeneous nuclear ribonucleoprotein D-like | 14.4 | 0.01045 |
| *58* | Dlst | Dihydrolipoyllysine-residue succinyltransferase component of 2-oxoglutarate dehydrogenase complex, mitochondrial | 14.3 | 0.03672 |
| *59* | Chn2 | Beta-chimaerin | 14.2 | 0.00293 |
| *60* | Kiaa1324 | UPF0577 protein KIAA1324 | 13.3 | 0.00003 |
| *61* | Kif2c | Kinesin-like protein KIF2C | 13.1 | 0.04959 |
| *62* | Snap25 | Synaptosomal-associated protein 25 | 13.0 | 0.01904 |
| *63* | Psmd12 | 26S proteasome non-ATPase regulatory subunit 12 | 12.7 | 0.00396 |
| *64* | Pgrmc2 | Membrane-associated progesterone receptor component 2 | 12.7 | 0.01721 |
| *65* | Rlbp1 | Retinaldehyde-binding protein 1 | 12.5 | 0.03284 |
| *66* | Nipsnap1 | Protein NipSnap homolog 1 | 12.5 | 0.03524 |
| *67* | Prdx6 | Peroxiredoxin-6 | 11.9 | 0.01381 |
| *68* | Uba1 | Ubiquitin-like modifier-activating enzyme 1 | 11.9 | 0.02459 |
| *69* | Stx12 | Syntaxin-12 | 11.8 | 0.00740 |
| *70* | Cadps | Calcium-dependent secretion activator 1 | 11.7 | 0.00060 |
| *71* | Crk | Adapter molecule crk | 11.7 | 0.03990 |
| *72* | Tubb2a | Tubulin beta-2A chain | 11.5 | 0.03054 |
| *73* | Appl1 | DCC-interacting protein 13-alpha | 11.3 | 0.02316 |
| *74* | Amph | Amphiphysin | 11.2 | 0.00370 |
| *75* | Prdx3 | Thioredoxin-dependent peroxide reductase, mitochondrial | 11.0 | 0.01328 |
| *76* | Nptn | Neuroplastin | 10.6 | 0.03883 |
| *77* | Impdh1 | Inosine-5'-monophosphate dehydrogenase 1 | 9.9 | 0.02426 |
| *78* | Psme3ip1 | PSME3-interacting protein | 8.2 | 0.00175 |
| *79* | Cs | Citrate synthase, mitochondrial | 8.2 | 0.03054 |
| *80* | Hprt1 | Hypoxanthine-guanine phosphoribosyltransferase | 8.1 | 0.00054 |
| *81* | Chchd3 | MICOS complex subunit Mic19 | 7.5 | 0.03564 |
| *82* | Hpca | Neuron-specific calcium-binding protein hippocalcin | 7.5 | 0.03284 |
| *83* | Pea15 | Astrocytic phosphoprotein PEA-15 | 7.0 | 0.04009 |
| *84* | Rpsa | 40S ribosomal protein SA | 7.0 | 0.04053 |
| *85* | Gstp2 | Glutathione S-transferase P 2 | 6.2 | 0.00048 |
| *86* | Rab3a | Ras-related protein Rab-3A | 6.2 | 0.03564 |
| *87* | Ptgfrn | Prostaglandin F2 receptor negative regulator | 6.1 | 0.00523 |
| *88* | Snrpa1 | U2 small nuclear ribonucleoprotein A' | 5.9 | 0.02217 |
| *89* | Sae1 | SUMO-activating enzyme subunit 1 | 5.8 | 0.00483 |
| *90* | Pygl | Glycogen phosphorylase, liver form | 5.6 | 0.00103 |
| *91* | Glud1 | Glutamate dehydrogenase 1, mitochondrial | 5.5 | 0.04879 |
| *92* | Sptbn1 | Spectrin beta chain, non-erythrocytic 1 | 4.8 | 0.00005 |
| *93* | Ccdc90b | Coiled-coil domain-containing protein 90B, mitochondrial | 4.8 | 0.00008 |
| *94* | Atp1a1 | Sodium/potassium-transporting ATPase subunit alpha-1 | 4.7 | 0.00995 |
| *95* | Eef1a2 | Elongation factor 1-alpha 2 | 4.4 | 0.03424 |
| *96* | Qars1 | Glutamine--tRNA ligase | 4.3 | 0.04879 |
| *97* | Acp1 | Low molecular weight phosphotyrosine protein phosphatase | 4.3 | 0.04111 |
| *98* | Ppp2cb | Serine/threonine-protein phosphatase 2A catalytic subunit beta isoform | 4.2 | 0.00483 |
| *99* | Ube2v2 | Ubiquitin-conjugating enzyme E2 variant 2 | 4.2 | 0.00740 |
| *100* | Lmna | Prelamin-A/C | 4.2 | 0.00654 |
| *101* | Psmd13 | 26S proteasome non-ATPase regulatory subunit 13 | 4.1 | 0.00043 |
| *102* | Smc1a | Structural maintenance of chromosomes protein 1A | 4.1 | 0.00704 |
| *103* | Psmd2 | 26S proteasome non-ATPase regulatory subunit 2 | 3.9 | 0.02445 |
| *104* | Eif2s2 | Eukaryotic translation initiation factor 2 subunit 2 | 3.8 | 0.04797 |
| *105* | Eif3e | Eukaryotic translation initiation factor 3 subunit E | 3.7 | 0.04220 |
| *106* | Ncl | Nucleolin | 3.7 | 0.02108 |
| *107* | Erlin2 | Erlin-2 | 3.6 | 0.02513 |
| *108* | Aifm1 | Apoptosis-inducing factor 1, mitochondrial | 3.5 | 0.04051 |
| *109* | Nop53 | Ribosome biogenesis protein NOP53 | 3.4 | 0.00498 |
| *110* | Cse1l | Exportin-2 | 3.4 | 0.00621 |
| *111* | Acly | ATP-citrate synthase | 3.2 | 0.04202 |
| *112* | Arf4 | ADP-ribosylation factor 4 | 3.2 | 0.04959 |
| *113* | Igsf3 | Immunoglobulin superfamily member 3 | 3.2 | 0.00999 |
| *114* | Hk1 | Hexokinase-1 | 3.1 | 0.00112 |
| *115* | Cct3 | T-complex protein 1 subunit gamma | 3.0 | 0.01604 |
| *116* | Arpc2 | Actin-related protein 2/3 complex subunit 2 | 3.0 | 0.00897 |
| *117* | Lrrc59 | Leucine-rich repeat-containing protein 59 | 3.0 | 0.04202 |
| *118* | Pfkm | ATP-dependent 6-phosphofructokinase, muscle type | 2.8 | 0.00740 |
| *119* | Cct6a | T-complex protein 1 subunit zeta | 2.8 | 0.02426 |
| *120* | Uba3 | NEDD8-activating enzyme E1 catalytic subunit | 2.5 | 0.01698 |
| *121* | Plec | Plectin | 2.5 | 0.04343 |
| *122* | Rps6 | 40S ribosomal protein S6 | 2.5 | 0.01160 |
| *123* | Atp6v0a1 | V-type proton ATPase 116 kDa subunit a isoform 1 | 2.2 | 0.03969 |
| *124* | Psmc2 | 26S proteasome regulatory subunit 7 | 2.2 | 0.03284 |
| *125* | Ipo5 | Importin-5 | 2.2 | 0.02184 |
| *126* | Tcp1 | T-complex protein 1 subunit alpha | 2.0 | 0.03564 |
| *127* | Ift122 | Intraflagellar transport protein 122 homolog | 2.0 | 0.03120 |
| *128* | Eftud2 | 116 kDa U5 small nuclear ribonucleoprotein component | 2.0 | 0.01964 |
