# Supplementary material for: Inceptor facilitates acrosomal vesicle formation in spermatids and is required for male fertility
Source: Front Cell Dev Biol. 2023 Aug 24;11:1240039. doi: 10.3389/fcell.2023.1240039 (PMC10483240; doi:10.3389/fcell.2023.1240039)
Supplement: Supplementary file 4 [file Table3.docx]

**Supplementary Figure S1.** Inceptor expression and *Iir^-/-^* mouse line generation. (A) Cell type-specific *Iir* expression (normalized transcripts per million TPM) in Human Protein Atlas scRNA-seq data. Shown are the top 20 cell types with highest *Iir* expression. Cell type groups, sharing common functional features, are shown in different colors. (B) Representative immunostaining confocal images of inceptor expression in various tissues. The image brightness setting is not maintained between images. The images of the stomach, salivary gland, and colon are maximum-intensity projections. Scale bar 25 μm. (C) schematic representation of the *Iir^-^* allele generation in mice. (D) percentage of the genotypes at weaning age of litters from *Iir^+/-^* intercrosses, n = 132. (E) Western blot of testis lysates from *Iir^+/+^* and *Iir^-/-^* mice.

**Supplementary Figure S2.** *Iir^-/-^* mice show normal testis gross morphology. (A) Body weight, (B) testis weight, and (C) caudal sperm count of 9–12-week-old *Iir^+/+^* and *Iir^-/-^* mice. (D) testis (T) and epididymis (epi) of *Iir^+/+^* and *Iir^-/-^* mice. Tick marks 1 mm. (E) Representative immunofluorescence images of the germ cells (DDX4) and Sertoli cells (GATA-4) in the seminiferous tubules. Image brightness has been adjusted to match DAPI intensity between images. Scale bar 50 μm. (F) Quantification of seminiferous tubule diameter at the narrowest section. Each dot represents a tubule measurement from a total of 87 and 66 tubules in *Iir^+/+^* and *Iir^-/-^* testes sections, respectively, n = 3 for both genotypes.

**Supplementary Figure S3.** *Iir^-/-^* spermatids have morphological defects in later stages of development. (A-C) Electron micrographs of *Iir^+/+^* elongated spermatid development and (D-F) *Iir^-/-^* elongated spermatid development. Scale bar 2 μm.

**Supplementary Figure S4.** Tree map of the reduction of GO:BP terms of the inceptor interactome. Schematic representation of the reduction of GO:BP terms into eleven parental terms.

**Supplementary Table 1.** Data used for plotting the log2(FC) and *p* value of the testis proteome. The *p* value was adjusted according to Hochberg. The hits are listed by descending -log10(*p* value).

**Supplementary Table 2.** Candidate interactors of inceptor from the mass spectrometry analysis. Arranged from top to bottom by descending fold change compared to a co-IP with a control antibody. The candidates have been filtered for *q* < 0.05, fold change > 2, and < 60 % missing values.
